# Understanding determinants related to farmers’ protective measures towards pesticide exposure: A systematic review

**DOI:** 10.1371/journal.pone.0298450

**Published:** 2024-02-15

**Authors:** Mehdi Kangavari, Mahsa Sarvi, Maryam Afshari, Shahnaz Maleki

**Affiliations:** 1 Department of Occupational Health and Safety, School of Public Health and Safety, Shahid Beheshti University of Medical Sciences, Tehran, Iran; 2 Department of Public Health, Hamadan University of Medical Sciences, Hamadan, Iran; 3 Social Determinants of Health Research Center, Hamadan University of Medical Sciences, Hamadan, Iran; 4 Research Center for Health Sciences, Hamadan University of Medical Sciences, Hamadan, Iran; Canakkale Onsekiz Mart University, TURKEY

## Abstract

**Objective:**

Pesticide poisoning is the main cause of adverse effects and mortality worldwide. Protective measures can reduce the intensity of the effects of pesticides on the health of farmers. Numerous cross-sectional studies have been conducted on the determinants of performing protective measures to reduce exposure to pesticides, but there is no systematic study that comprehensively examines the impact of these factors. Therefore, the aim of this study is to identify existing studies on the determinants of effective protective measures to reduce exposure to pesticides among farmers.

**Methods:**

In this systematic review, studies were obtained from PubMed, Web of Science and Scopus databases using a search strategy that covered articles from the first years of database design to April 20, 2023. The inclusion and exclusion criteria were based on the PICOs criteria. The study included cross-sectional studies that measured the implementation of protective measures using objective or valid subjective tools. The data were extracted and analyzed based on several criteria and ecological levels. The Ecological Model of Health Behavior was used to classify the determinants that affect the performance of protective behaviors. The National Heart, Lung and Blood Institute (NHLBI) has developed a quality assessment tool for studies.

**Results:**

A total of 39 studies were ultimately selected for inclusion in this analysis. Many of these studies were conducted in developing countries. The most important factors that have an impact on protective measures include a variety of socio-demographic characteristics (age, gender, level of education, income, farming experience, experience of using pesticides), individual level (knowledge, attitude, risk perception, intention), interpersonal level (subjective norms), organizational level (education), and public policy level (government attention, health costs, governmental extension services). The quality of most studies was fair.

**Conclusions:**

Research indicates that several factors influence the use of personal protective equipment and safe behaviors when handling pesticides. These include farmers’ education level, knowledge, and attitudes towards safety measures. Environmental factors such as access to information, extension services, training programs, and media coverage can also help minimize exposure to pesticides.

## 1. Introduction

Pesticides play a crucial role in controlling harmful or destructive pest species in crops, including insects, weeds, and disease-causing agents [1]. Therefore, the use of pesticides is currently a key strategy in pest management to ensure food supply and distribution worldwide [2]. However, reports indicate that some pesticides pose serious threats to human health and the environment [3]. Several studies have shown that improper use of pesticides can increase the incidence of poisoning, disability, and death associated with pesticide exposure [4,5].

Pesticide poisoning is the main cause of adverse health effects and mortality worldwide. It is estimated that the annual incidence of pesticide poisoning among agricultural pesticide users in developing countries is about 18.2 cases per 100,000 people [6]. However, estimating the actual incidence of pesticide poisoning among farmers in developing countries is difficult [7]. Therefore, the use of pesticides has increased health risks for farmers [8].

The actions such as avoiding the health hazards of pesticides, adopting protective behaviors (PBs), using personal protective equipment (PPE), and appropriate use of pesticides during handling, transportation, mixing, and spraying are recognized as protective measures that can reduce the intensity of the effects of pesticides on the health of farmers [8,9]. Most farmers do not consider the use of safety measures that can reduce the risk of pesticide poisoning [10]. Therefore, identifying the effective factors on protective measures when using pesticides among farmers is essential [11].

A review of previous studies in the field of safety shows that various factors can influence protective measures among farmers when faced with pesticide poisoning [12]. Factors such as age, education level, farming experience [13], perceived risk, awareness [9,10], attitudinal and belief variables [11], perceived barriers, facilitators, health expectations, social norms, emotions, physiological arousal, and intention are among the factors that determine the implementation of protective measures by farmers during pesticide use [14].

Many farmers who suffer from pesticide poisoning often do not report it due to concerns about losing their job, high costs, and lack of access to health care [6]. Additionally, health care professionals often cannot accurately diagnose pesticide poisoning. Therefore, there is less reporting of pesticide poisoning among farmers [15]. Furthermore, the status of protective measures and their effective determinants in a comprehensive study to reduce poisoning and exposure to pesticides is unclear [16]. Numerous cross-sectional studies have been conducted on the determinants of performing protective measures to reduce exposure to pesticides, but there is no systematic study that comprehensively examines the impact of these factors. Therefore, the aim of this study is to identify existing studies on the determinants of effective protective measures to reduce exposure to pesticides among farmers.

## 2. Materials and methods

The present study was a systematic review, and the PRISMA checklist was used to improve the transparency and quality of reporting.

### 2.1 Data collection

#### Search databases

The articles were obtained from the PubMed, Web of Science, and Scopus databases using a search strategy developed by one of the researchers. In this search strategy, the keywords present in Appendix A were used. The search strategy was for articles searched from the first years of database design to April 20, 2023. Modifications were made to this search strategy according to the differences in search capabilities of each database.

#### Study criteria

Based on the PRISMA criteria, the inclusion and exclusion criteria for this study were determined.

Inclusion criteria

Study type: Cross-sectional studies were considered for inclusion in this study.

Study topic: Studies that investigated the determinants and factors affecting the use of protective measures (PBs or PPE) among farmers when dealing with pesticides were examined.

Study population: Farmers who used pesticides in the agricultural sector.

Study outcomes: The outcome of interest was the implementation of protective measures (PBs or use of PPE), which was measured using objective criteria (observation) or valid subjective tools (self-reporting).

Timeframe: Studies published from the earliest years available in the searched databases were included.

Language: Studies published in all languages were considered.

Location: Studies conducted in all countries and on both large and small farms were reviewed.

Exclusion criteria

The absence of appropriate format in articles, inaccurate mention of sample size and characteristics, study design, special studies (such as letters to the editor, comments), review articles, structured reviews, meta-analyses, conference presentations, qualitative studies, various intervention studies, cohort and case-control studies, as well as agricultural and floricultural workers, greenhouse workers, women, and children in rural areas, were considered as exclusion criteria.

#### Identification of studies

All retrieved articles from the selected databases were organized and screened using EndNote software. The search for articles from the databases was conducted by one of the researchers to minimize errors.

### 2.2 Data extraction

#### Screening studies

After removing any duplicates, the titles and abstracts of the articles are assessed to determine their eligibility. The full text of eligible articles and relevant data are then extracted and recorded in a data extraction table. This process is carried out independently by two researchers. In case of disagreement on the remaining articles, a third researcher will review and provide their opinion to resolve the conflict. Additionally, the list of references used in the remaining and relevant articles is also reviewed to ensure completeness.

#### Data extraction

The data were extracted based on the following criteria: first author’s name, year of study, location of study, study objective, journal name, sampling technique, sample size, determinants examined in the studies, model or theory used in the studies, and study results. Additionally, the determinants were evaluated as independent variables at five ecological levels, and their impact on the dependent variable, which includes performing PBs or using PPE, was examined in the study. Demographic and background characteristics were also examined in the data extraction process.

#### Ecological levels

In this study, the Ecological Model of Health Behavior (EMHB) was used to classify determinants that affect the performance of protective behaviors [17]. The first level of the model is the individual level, which is related to the knowledge, attitudes, and skills that are directly related to the individual. The second level is the interpersonal level, which includes the exchanges and interactions within an individual’s network. This includes primary relationships, such as family and close friends, as well as secondary groups that are larger and more extensive. The third level is the organizational level focused on social institutions that act as official authorities and provide public and accepted goals. The fourth level is the community level, which includes relationships that organizations create with each other. These relationships are often found in coalitions. Finally, the fifth level is the policy and public policy level that is adopted by local and national governments. Ideally, when integrating and using this model, all five levels are taken into account.

#### Risk of bias assessment

The quality assessment tool for cross-sectional studies available from National Heart, Lung and Blood Institute (NHLBI), USA was used for quality assessment [18]. This tool consists of 14 criteria that cover various aspects of the study design, methods, and analysis, such as the research question, the study population, the exposure and outcome measures, the confounding variables, and the statistical methods. For each criterion, researchers can answer yes, no, or other (CD, NR, NA), depending on whether the study met the criterion, did not meet the criterion, or they cannot determine, not reported, or not applicable. Researchers can also provide a quality rating (good, fair, or poor) for each study based on their overall assessment of the criteria. Quality is rated on a scale of 0 to 2, where 0 represents poor quality, 1 represents fair quality, and 2 represents good quality. The rating is determined based on the number of questions answered correctly out of a total of 14 questions. A score of 0–4 out of 14 questions corresponds to a rating of 0 (poor), a score of 5–10 out of 14 questions corresponds to a rating of 1 (fair), and a score of 11–14 out of 14 questions corresponds to a rating of 2 (good). NA stands for “not applicable” and NR stands for “not reported”.

## 3. Results

### Identification of studies

**Fig 1:**
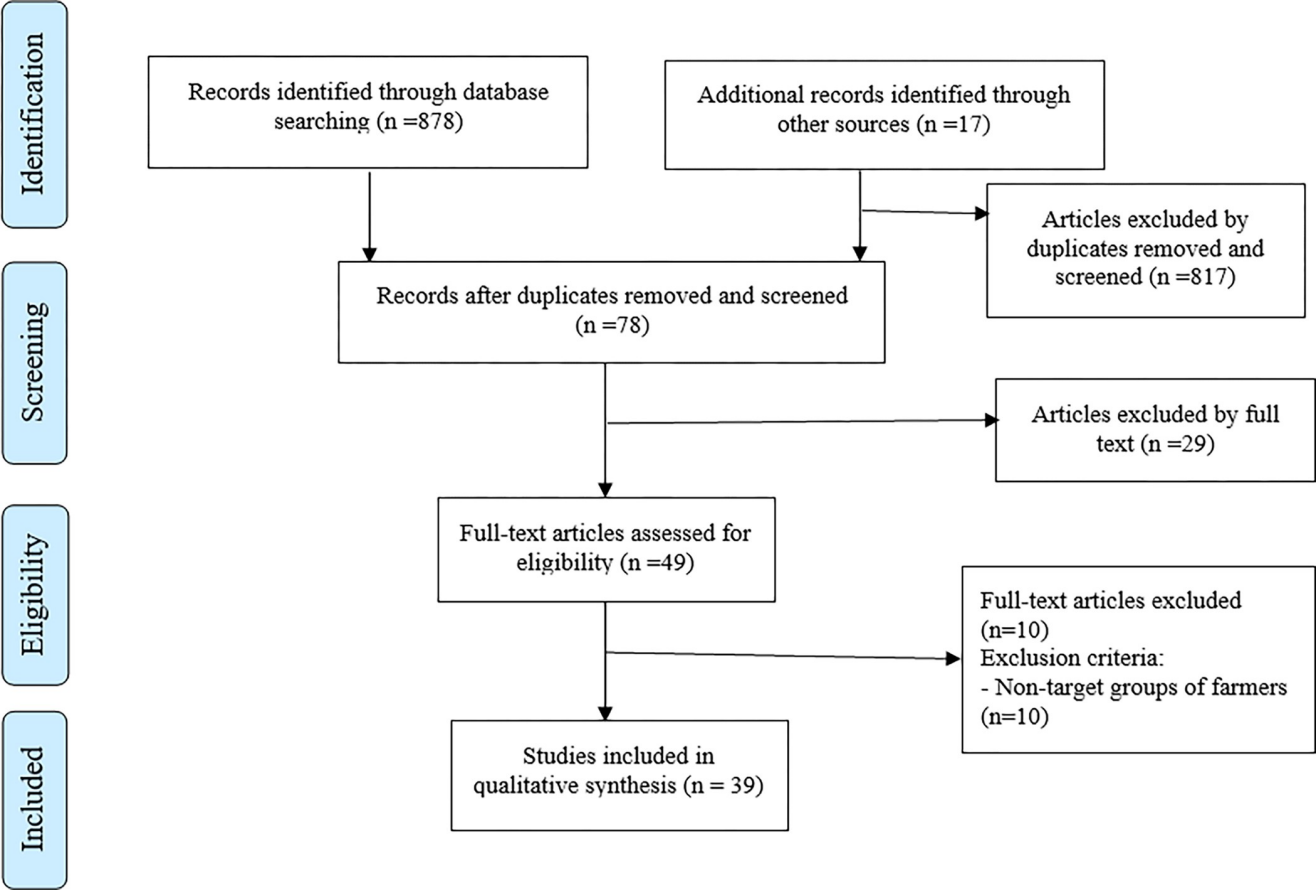
Flow diagram for the identification, screening, eligibility, and inclusion of studies.

Through electronic search engines and strategies, a total of 895 studies were identified. During the initial evaluation of duplicate and title, 817 of these studies were excluded as they did not meet the inclusion criteria (see Fig 1). Twenty nine articles were excluded during the evaluation of abstracts, resulting in a total of 49 studies for full-text assessment. After further assessment, a final selection of 39 studies was made for inclusion in the analysis.

### Study characteristics

**Fig 2:**
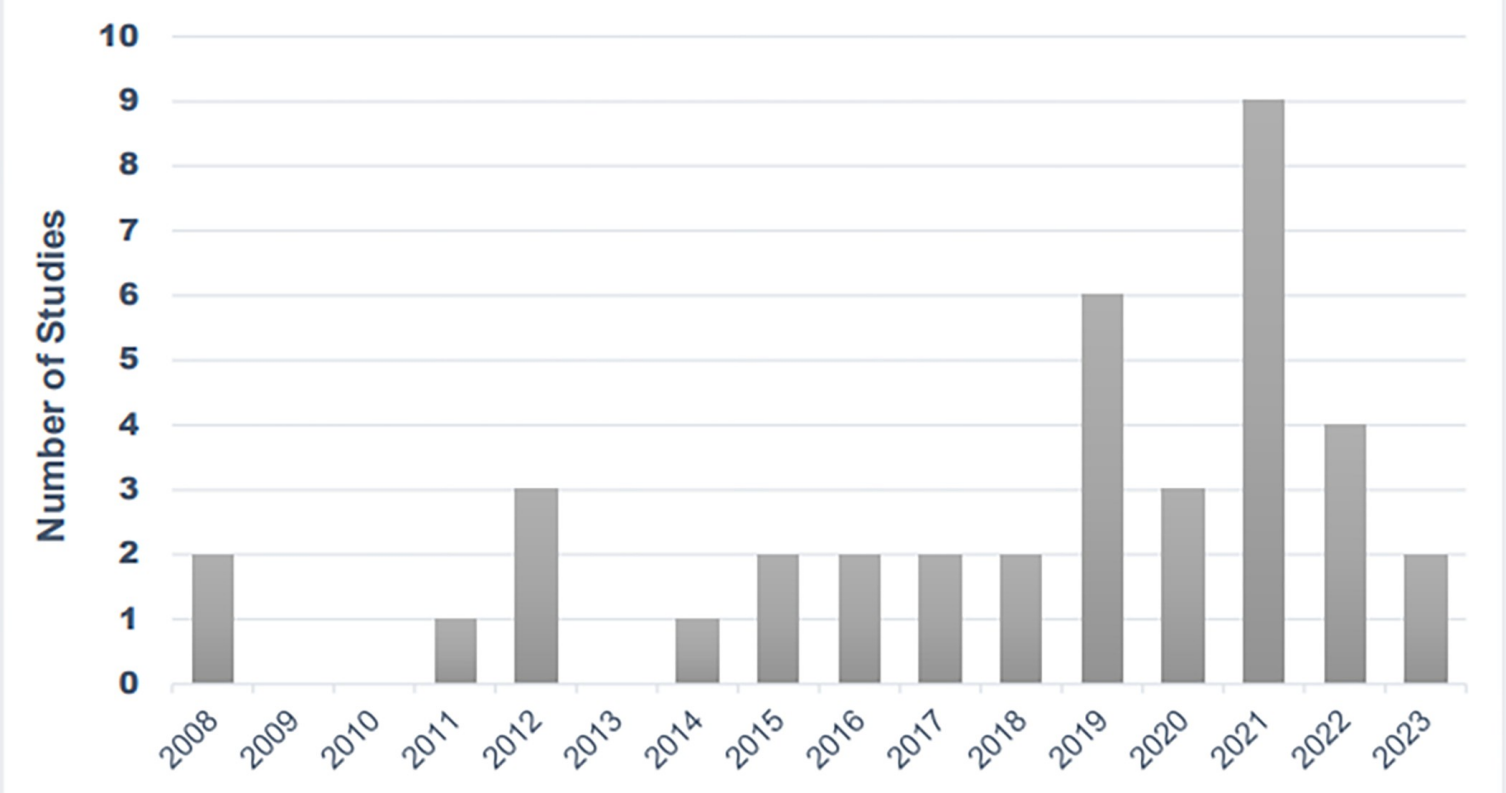
Studies for farmer conducted from 2008 until 2023.

Studies have been conducted on farmers from 2008 until 2023 (Fig 2).

The articles included in the analysis were based on studies conducted in the Australia (n = 1) [19], Iran (n = 4) [12,14,20,21], Canada (n = 1) [22], China (n = 1) [23], Uganda (n = 3) [24–26], Thailand (n = 3) [27–29], Egypt (n = 1) [30], Brazil (n = 2) [31,32] Nepal (n = 3) [33–35], Pakistan (n = 3) [36–38], Italy (n = 1) [39], Turkey (n = 1) [40], India (n = 1) [41], Malaysia (n = 1) [42], Ghana (n = 1) [43], Morocco (n = 2) [44,45], Nigeria (n = 2) [46,47], Vietnam (n = 1) [48], Philippine (n = 1) [49], Costa Rica and Uganda (n = 1) [50], and Ethiopia (n = 5) [51–55]. Among the 39 articles obtained in this study, sampling techniques included simple random sampling (n = 12) [19,22,25,26,28,30,38,41,48,52,53,55], quota sampling (n = 3) [34,36,44], systematic random sampling (n = 2) [29,35], stratified random sampling (n = 1) [54], convenience sampling (n = 3) [21,31,39], purposive sampling (n = 1) [42], snowball sampling (n = 4) [32,46,47,50], not clear (n = 1) [44], and some were multi stage sampling (n = 12) [12,14,20,23,24,27,33,37,40,43,49,51]. The total number of samples was low. Less than 350 samples were used in twenty three studies [12,20,21,24–28,30–34,36–39,41–43,45,47,53].

### Theoretical framework usage

A theoretical framework was applied in only four articles (10.2%) that were included. The integrated agent centered (IAC) framework [14], precede-proceed framework [22], trans-theoretical model (TTM) [28], and theory of planned behavior (TPB) [12] were the theories that were used (Table 1).

**Table 1:** Determinants related to farmers’ protective measures towards pesticide exposure.

Authors/ Country	Objective of the study	Journal	Sampling technique	Sample size	Determinants examined in studies						Theoretical framework	Results	Quality Rating
					Demographic	Individual level	Interpersonal level	Organizational level	Community level	Public policy level			
Afshari et al. (2019)/ Iran [14]	To identify the underlying factors influencing farmers’ protective behaviors and use of personal protective equipment during the exposure to pesticides	Workplace Health & Safety	First cluster the random sampling	N = 474	Sex, age, education, marital status, income, farming years, spraying years, ownership type of farm land	Knowledge, perceived barriers, facilitators, social outcome expectations, health outcome expectations, expected value of consequences, affect, physiological arousal, habit, intention	Social norms	-	-	-	Integrated Agent Centered (IAC) framework	- Positive impact on PBs: Physiological arousal, intention, habit, knowledge, perceived barriers, facilitators - Significant effect on the use of PPE: physiological arousal and habit	Good
MacFarlane et al. (2008)/ Australia [19]	To investigate patterns of use of personal protective equipment to reduce pesticide exposure	Occupational and Environmental Medicine	Simple random sampling	N = 1102	Age, education, smoking and alcohol consumption	-	-	Farm training	-	-	-	- Positively associated with PPE: farm chemical training, younger age, alcohol consumption, education	Fair
Nicol et al. (2008)/ Canada [22]	To investigate the prevalence of wearing personal protective equipment or by implementing alternative pest control techniques	Journal of Occupational and Environmental Hygiene	Simple random sampling	N = 750	Age, gender, ethnicity, education, marital status	Knowledge, attitudes, risk perception	Reinforcing factors	Enabling factors	-	-	Precede-Proceed framework	- Significantly associated with PPE use: ethnicity, reinforcing characteristics, perception of pesticide risk	Fair
Zhang et al. (2011)/ China [23]	To investigate the prevalence and risk factors of acute work-related pesticide poisoning	BMC Public Health	First classification and then census	N = 910	Gender, education, age, application time	Knowledge	-	-	-	-	-	- Positive impact on PBs: gender of male, older applicators, safety knowledge	Good
Gaber et al. (2012)/ Egypt [30]	To investigate knowledge and behaviors of farmers related to pesticide use and their relation to educational level and health locus of control	Journal of Occupational Medicine and Toxicology	Simple random sampling	N = 335	Age, marital status, educational level, income, medical history of chronic diseases	Knowledge, locus of control	-	-	-	-	-	- Positive impact on PBs: higher of education level, knowledge and locus of control	Fair
Markmee et al. (2012)/ Thailand [27]	To identify and assess the health effects of insecticide use among rice farmers	Journal of Health Research	Multi-stage sampling	N = 182	Gender, age, marital status, educational level, income, farm size	Knowledge, attitude	-	Trained in safe pesticide use	-	-	-	- Positive impact on PBs: Knowledge, attitude and trained in safe pesticide use	Fair
Pasiani et al. (2012)/ Brazil [31]	To evaluated the knowledge, attitudes and practices regarding pesticide use and the levels of exposure of farmers	International Journal of Environmental Research and Public Health	Convenience sampling	N = 112	Age, education, years of pesticide use, consumption of alcohol	Knowledge, attitudes	-	-	-	-	-	- Positive impact on PPEs: Knowledge, attitudes	Fair
Oesterlund et al. (2014)/ Uganda [24]	To describe pesticide use among small-scale farmers	African Health Sciences	The first convenience and then snowballing	N = 317	Age, gender, marital status, educational level, number of times of spraying	Knowledge, attitude	-	-	-	-	-	- Positive impact on PBs: knowledge, attitude and number of times of spraying	Fair
Okonya et al. (2015)/ Uganda [25]	To promote proper and safe pesticide-handling practices by providing data needed to guide pesticide regulation policy and training for extension staff and farmers	BioMed Research International	Simple random sampling	N = 204	Sex, age, education level	Knowledge, attitudes	-	-	-	Sources of Pesticide Information	-	- Positive impact on PBs: knowledge, attitude and sources of pesticide information	Fair
Wichai et al. (2015)/ Thailand [28]	To develop a readiness scale in changing behavior of using pesticides of farmers	Journal of the Medical Association of Thailand	Simple random sampling	N = 247	Income	Confidence, eagerness to learn, risk perception, levels of readiness to change, knowledge	-	-	Social	-	Trans-Theoretical Model (TTM)	Positive impact on PBs: income, social, confidence, eagerness to learn, risk perception, levels of readiness to change, knowledge	Fair
Gesesew et al. (2016) / Ethiopia [51]	To assess knowledge, attitudes and experiences of previous pesticide exposure, and related health problems	Plos One	Multi stage sampling	N = 796	Sex, age, educational status, presence of children, presence of pregnant, cultivated agricultural products, cigarette smoking	Knowledge, attitudes	-	-	-	-	-	Positive impact on PBs and PPE: knowledge, attitudes	Fair
Khanal et al. (2016)/ Nepal [33]	To identify the prevailing practices of pesticide use and factors affecting the handling of pesticides	Environmental Health Insights	Multistage sampling	N = 125	Age, sex, land owned, education, marital status, duration use of pesticides	Knowledge	-	-	-	-	-	Positive impact on PBs and PPE: knowledge, male and literate	Fair
Muleme et al. (2017)/ Uganda [26]	To use qualitative and quantitative data on pesticide usage to test this linear relationship, identify associated context specific factors	Frontiers in Public Health	Simple random sampling	N = 167	Age, sex, marital status, education level, farming type, land ownership, farming experience	Knowledge, attitude	-	-	-	-	-	Positive impact on PBs: knowledge, attitudes	Fair
Saeed et al. (2017)/ Pakistan [36]	To assess public awareness of pesticide risks and determine the levels of exposure to organochlorine pesticides	Science of the Total Environment	Quota sampling	N = 179	Gender, age, education	Knowledge	-	-	-	-	-	Positive impact on PBs and PPE: knowledge	Fair
Riccò et al. (2018)/ Italy [39]	To Assesse knowledge, attitudes and practices regarding pesticide handling and related health problems	Journal of Preventive Medicine and Hygiene	Convenience sampling	N = 260	Gender, smoking status, age, education level, type of crop grown, years of pesticide use	Knowledge, attitudes	-	-	-	-	-	Positive impact on PBs and PPE: knowledge, attitudes and years of pesticide use	Fair
Sharafi et al. (2018)/ Iran [20]	To investigate the knowledge, attitude, and behavior of farmers about the pesticide use	Science of the Total Environment	First cluster and then random sampling	N = 311	Age, gender, education, agriculture experience, crops produced, income, family size	Knowledge, attitudes	-	Pesticide training	-	-	-	Positive impact on PBs and PPE: knowledge, attitudes, age, education level, and pesticide training	Fair
Lamichhane et al. (2019)/ Nepal [34]	To explore the health effects of Pesticides among agricultural farmers	Journal of Nepal Health Research Council	Quota sampling	N = 300	Age, gender, education, size of family, years of working on farm, number of spraying time	Knowledge	-	Training on safe handling of pesticide	-	-	-	Positive impact on PBs and PPE: knowledge, working experience	Fair
Mequanint et al. (2019)/ Ethiopia [52]	To assess pesticide handling and storage practice, and its associated factors among farmers engaged in irrigation.	BMC Research Notes	Simple random sampling	N = 409	Sex, age, religion, marital status, level of education, income, family size	Knowledge, attitude	-	-	-	-	-	Positive impact on PBs: Knowledge, attitude and educational status	Fair
Quansah et al. (2019)/ Ghana [43]	To address knowledge, pesticide handling practices, and protective measures related to pesticide use	Environmental Monitoring and Assessment	Multi stage sampling	N = 310	Age, marital status, gender, level of education	Knowledge	-	-	-	-	-	Positive impact on PBs and PPE: knowledge	Fair
Rezaei et al. (2019)/ Iran [12]	To understand Iranian farmers’ intention to apply personal protective equipment	Safety Science	Multi stage sampling	N = 322	Age, education, family size, income	Attitude, perceived behavioral control, intention, perceived susceptibility, perceived severity	Subjective norms	-	-	-	Theory of Planned Behavior (TPB)	Positive impact on PBs and PPE: Attitude, perceived behavioral control, intention, perceived susceptibility, perceived severity and subjective norms	Good
Cevik et al. (2020)/ Turkey [40]	To investigating the relationship between the sociodemographic characteristics of farmers and their knowledge and attitudes towards pesticide use	Rocz Panstw Zakl Hig	Multi-stage sampling	N = 2100	Age, marital status, education level, family size, income, length of time spent in farming	Knowledge, attitudes	-	-	-	-	-	Positive impact on PBs and PPE: Knowledge, attitudes and age	Fair
Jambari et al. (2020)/ Malaysia [42]	To investigate the level of knowledge, attitude and practice on pesticide exposure	Malaysian Journal of Medicine and Health Sciences	Purposive sampling	N = 144	Gender, marital status, age, race, educational level	Knowledge, attitude	-	-	-	-	-	Positive impact on PBs and PPE: Knowledge, attitudes and gender	Fair
Staudacher et al. (2020)/ Costa Rica and Uganda [50]	To aim at identifying commonalities and differences in pesticide use practices	Environmental Health Insights	Snowball sampling	N = 602	Age, sex, education, marital status, years working in agriculture, handling/spraying pesticides, income	Knowledge, attitude	-	-	-	-	-	Positive impact on PBs and PPE: Knowledge, attitudes and income	Fair
Bakhtawer et al. (2021)/ Pakistan [37]	To access the current knowledge and common practices of farmers about the use of insecticides	PLoS ONE	Multi stage sampling	N = 300	Age, education level	Knowledge	-	-	-	-	-	Positive impact on PBs and PPE: Knowledge and education level	Fair
Ben Khadda et al. (2021)/ Morocco [44]	To assess the attitudes, knowledge, and practices regarding pesticide use	International Journal of Environmental Research and Public Health	Quota sampling	N = 526	Age, education level, years of experience in agriculture	Knowledge, attitude, decision-making	-	Pesticide training	-	-	-	Positive impact on PBs and PPE: Knowledge, attitude, decision-making, Pesticide training and education level	Fair
Berni et al. (2021)/ Morocco [45]	To assess farmers’ pesticide safety behavior	Sustainable Production and Consumption	Not clear	N = 232	age, education, gender, farming experience, type of land ownership	Knowledge, perceived risk, health consequences	Subjective norms	Pesticide training	-	-	-	Positive impact on PBs: Knowledge, perceived risk, health consequences and subjective norms	Fair
Buralli et al. (2021)/ Brazil [32]	To discuss the knowledge, attitudes, and regarding the impact of pesticides on health	Saude e Sociedade	Snowball sampling	N = 103	length of exposure; age	knowledge, attitudes	-	-	-	-	-	Positive impact on PPE: Knowledge, attitude	Fair
Kafle et al. (2021)/ Nepal [35]	To assess the practice of chemical pesticide use and associated factors	International Journal of Environmental Research and Public Health	Systematic random sampling	N = 790	Gender, age, ethnicity, education	Knowledge, risk perception, attitude	-	-	-	-	-	Positive impact on PBs and PPE: Knowledge, risk perception, attitude	Fair
Kangkhetkron et al. (2021)/ Thailand [29]	To survey the level of knowledge, attitude, and practice in terms of pesticide use	International Journal of Environmental Research and Public Health	Systemic random sampling	N = 680	Gender, age, education, duration of pesticide use	Knowledge, attitude	-	-	-	-	-	Positive impact on PBs and PPE: education, work experience, knowledge and attitude	Fair
Lari et al. (2021)/ India [41]	To evaluate farmers’ knowledge on pesticides handling	Archives of Environmental and Occupational Health	Simple random sampling	N = 217	Gender, age, education, farming experience	Knowledge	-	-	-	-	-	Positive impact on PBs and PPE: knowledge	Fair
Mehmood et al. (2021)/ Pakistan [38]	To examine determinants of farmers’ pesticide handling practices	Environmental Research	Simple random sampling	N = 307	Age, children under five years, farm size, income	Health effects	-	Pesticide training	-	Health costs	-	Positive impact on PBs and PPE: health costs, pesticide training and health effects	Fair
Nwadike et al. (2021)/ Nigeria [46]	To measure knowledge, attitudes, and practices related to pesticide application	Sustainability	Snowball sampling	N = 524	Age, gender, education status, smoking habit, farm size	Knowledge, attitude	-	-	-	-	-	Positive impact on PBs and PPE: educational level, age, years of farm practice experience, knowledge and attitude	Fair
Afata et al. (2022)/ Ethiopia [53]	To assess the prevalence of pesticide use and its occupational exposure among small-scale farmers	Environmental Health Insights	Simple random sampling	N = 249	Age, sex, marital status, educational level	Knowledge, attitude	-	-	-	-	-	Positive impact on PBs and PPE: educational level, age, knowledge and attitude	Fair
Galli et al. (2022)/ Vietnam [48]	To assess the patterns and relationships of official sustainable agriculture educational programs, pesticide safety knowledge, and practices	Journal of Occupational and Environmental Hygiene	Simple random sampling	N = 400	Sex, age, marital status, religion, educational status, experience spraying	Knowledge, attitude	-	Exposure to official educational programs	-	Governmental extension services	-	Positive impact on PPE: high education, exposure to official educational programs, knowledge, and governmental extension services	Good
Lu et al. (2022)/ Philippine [49]	To investigate the knowledge, attitude, and practices of farmers about pesticide	Acta Medica Philippina	Multi stage sampling	N = 387	Sex, marital status, educational	Knowledge, attitude	-	-	-	-	-	Positive impact on PBs and PPE: knowledge and attitude	Fair
Moda et al. (2022)/ Nigeria [47]	To measure knowledge, attitude, and practices related to pesticide storage, handling, application, and containers disposal	International Journal of Environmental Research and Public Health	Snowball sampling	N = 285	Age, gender, level of education, smoking habit, number of years working with pesticide	Knowledge, attitude	-	-	-	-	-	Positive impact on PBs and PPE: knowledge and attitude	Fair
Alebachew et al. (2023)/ Ethiopia [54]	To assess pesticide use safety practices and associated factors among farmers	PLoS ONE	Stratified random sampling	N = 430	Sex, age, marital status, religion, educational status, experience with pesticide spray, income, farm size	Knowledge, attitude	-	Trend of using pesticides and access to safety materials	Environmental factors	government attention, media coverage, enforcement of laws, guideline access	-	Positive impact on PBs and PPE: educational status, experience of pesticide spraying, knowledge of pesticide usage, access to safety materials, and ever having received training, government attention, media coverage, enforcement of laws, guideline access	Good
Lelamo et al. (2023)/ Ethiopia [55]	To assess pesticide use practice and its associated factors	Environmental Health Insights	Simple random sampling	N = 549	Age, education level, year of experience of utilization of pesticide	Knowledge, perceived importance, perception of risk	-	-	-	-	-	Positive impact on PBs and PPE: level of education, year of experience of utilization of pesticide, the place of pesticide bought, perceived importance perception of risk, knowledge	Fair
Rostami et al. (2019)/ Iran [21]	To evaluate the knowledge, attitude, and practice of pesticides use	Indian Journal of Occupational and Environmental Medicine	Convenience sampling	N = 262	Educational level, marital status, age, duration of pesticide use	Knowledge, attitude	-	-	-	-	-	Positive impact on PBs and PPE: knowledge, attitude	Fair

### Categorization of determinants

**Fig 3:**
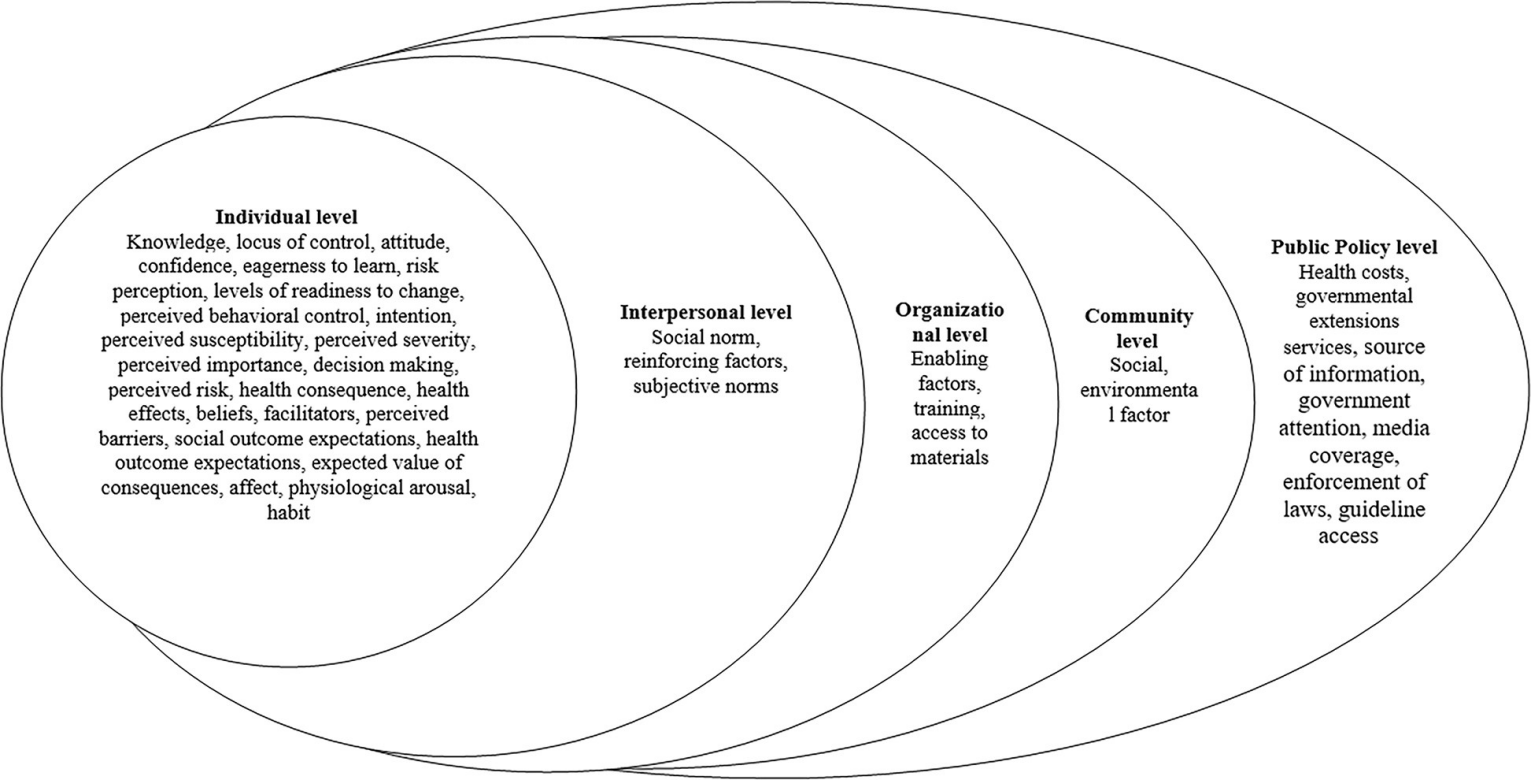
Ecological approach for classify determinants effect on protective measures.

The factors under investigation and the factors affecting the performance of protective measures when exposed to pesticides in farmers are shown in Table 1 and Fig 3.

1) Demographic examined in studies

All studies had taken into account demographic characteristics more or less. Except for two studies [28,29], other studies had taken age as one of the demographic characteristics into account. Twenty five articles had considered gender [14,22–26,29,33–36,39,41–43,45–54]. The level of education had been examined for the participants in Twenty nine articles [12,14,19–27,29–31,33–37,39–41,43–46,48–55]. Also, the marital status of the participants had been investigated in seventeen studies [14,21,22,24,26,27,30,33,40,42,43,48–50,52–54]. The income status of the farmers had also been asked in eleven articles [12,14,20,27,28,30,38,40,50,52,54]. The years of farming of the farmers was another characteristic that was examined in eight studies [14,20,34,40,41,44,45,50]. The duration of exposure with pesticides and the years of spraying crops were other items that had been asked from the farmers in twelve studies [14,23,24,29,31–34,39,47,48,50]. The ownership of the orchard or agricultural land was another item that had been investigated in four studies [14,26,33,45]. Also, race/ ethnicity had been reported in three studies [22,35,42] and religion in two articles [48,52]. The size of the farm [27,38,46,54] and family size [12,20,40,52] were also another factor that had been asked from the farmers. The consumption of smoking and alcohol by the farmers had also been reported in six studies [19,31,39,46,47,51]. The history of having a child and a pregnant woman had also been examined in two studies [38,51]. The type of crops in two studies [20,26,39,51] and the medical history of the farmers in one study [30] had been considered.

2) Ecological Model of Health Behavior examined in studies

Individual level: Almost all studies except for three studies had considered knowledge as a determinant of protective measures [12,19,38]. Also, most studies had examined attitude as a determinant of performing protective measures [12,20–22,24–27,29,31,32,35,39,40,42,46–54]. Risk perception/ perceived risk was the next determinant that had been investigated in five studies out of 39 studies [22,28,35,45,55]. The intention in two studies was considered to see what relationship it had with protective actions [12,14]. The locus of control [30], confidence, eagerness to learn, levels of readiness to change [28], health effects [38], perceived importance [55], decision making [44], and health consequences [45] each were investigated in one study. The perceived behavioral control, perceived susceptibility, and perceived severity were also only investigated in one study [12]. The determinants of perceived barriers, facilitators, social outcome expectations, health outcome expectations, expected value of consequences, affect, physiological arousal, and habit, in a study were investigated [14].

Interpersonal level: Reinforcing factors in one study were considered as a determinant of protective measures [22]. Also, subjective norms were considered in two studies [12,45]. Social norm was also investigated in another study [14].

Organizational level: In eight studies, education to farmers about pesticides and exposure to them through governmental and private organizations were considered as an organizational factor [19,20,27,34,44,45,48,54]. Enabling factors [22] and access to materials [54] were also each investigated in one study.

Community level: At this level, only studies had examined two social [28] and environmental factors [54] that could affect protective measures.

Public policy level: The determinants of government attention, media coverage, enforcement of laws, and guideline access, in a study were investigated [54]. In another study, governmental extensions services were considered for the impact on protective measures in exposure to pesticides [48]. Also, in one study, health costs [38] and in another study, the source of information dissemination [25] were examined.

### Determinants related to farmers’ protective measures

In the following, the variables that had a positive effect on protective measures and had increased the performance of these actions are mentioned and the determinants that had no relationship are not explained.

1) Demographic examined in studies

The age of farmers had a statistically significant association with protective behavior to reduce exposure to pesticides [19,20,23,40,46,53]. In three studies, gender had a statistically significant association with protective measures [23,33,42]. Also, in thirteen studies, the results showed that the level of education of farmers had a statistically significant association with protective measures [19,20,29,30,33,37,44,46,48,52–55]. The income of farmers also showed a statistically significant association with protective measures in two studies [28,50]. In three studies, farming experience [29,34,46] and in three studies, experience of using pesticides [24,39,55] showed a statistically significant association with protective measures. In one study, ethnicity [22] and in another study, alcohol consumption [19] had a significant statistical relationship with protective measures.

2) Ecological Model of Health Behavior examined in studies

Individual level: Out of 36 studies that examined knowledge, it was found that 35 studies had a statistically significant association with protective measures [14,20,21,23–37,39–55]. Out of 25 studies that worked on attitude, it was found that in three studies, no statistically significant association with protective measures was reported [12,20,21,24–27,29,31,32,35,39,40,42,44,46,47,49–53]. All five studies that used risk perception/ perceived risk reported that this construct could have a positive effect on protective measures [22,28,35,45,55]. Both studies that examined the effect of intention on protective measures reported a statistically significant association between the two [12,14]. The constructs of locus of control [30], confidence, eagerness to learn, levels of readiness to change [28], health effects [38], decision making [44], perceived importance [55], perceived behavioral control, perceived susceptibility, perceived severity [12], physiological arousal, habit perceived barriers, facilitators [14], and health consequences [45] had a statistically significant association with protective measures.

Interpersonal level: The constructs of reinforcing factors had a statistically significant association with protective measures [22]. Also, both studies that examined the effect of subjective norms on protective measures reported a statistically significant association between the two [12,45].

Organizational level: Out of eight studies that farmers had considered the factor of farmer education by organizations, seven studies confirmed the association between acquiring these educations and protective measures [19,20,27,38,44,48,54]. In addition, the access to materials had a statistically significant association with protective measures [54].

Public policy level: The determinants of government attention, media coverage, enforcement of laws, and guideline access, in a study were investigated [54], governmental extensions services [48], health costs [38], and source of information dissemination [25] had a statistically significant association on protective measures in exposure to pesticides.

### Quality assessment

The quality of most studies was fair, and only five studies had good quality in the assessment by researchers [12,14,23,48,54].

## 4. Discussion

In this study, after careful examination of articles by researchers, 39 studies were identified in the field of determining the effective determinants of protective measures to reduce exposure to pesticides. In this systematic review, most studies were conducted in developing countries. In developing countries, farmers are at high risk of exposure to pesticides due to having small-scale agricultural land and gardens, as well as a lack of protective equipment, climatic conditions, and excessive use of pesticides [56]. Contrary to our study, the systematic study by Afshari and et al (2021), which was conducted on interventional studies in the field of reducing exposure to pesticides in farmers, showed that most studies were conducted in developed countries, including the United States [57]. Wiedemann and colleagues in a systematic study showed that in developing countries, extension services cannot reach the entire agricultural community due to insufficient budget [58]. In wealthy and developed countries, strict regulations have been imposed for the use of pesticides, and exposure to toxic chemicals is prohibited or limited for farmers. While in developing countries, regulations and supervision of the use of pesticides are challenging and not implemented, and farmers are exposed to a large extent to toxic and vulnerable substances [59,60]. So that, 1% increase in crop production per hectare is associated with a 1.8% increase in pesticide use per hectare. However, as countries reach higher levels of economic development, the growth in pesticide use intensity decreases [61].

The current study found that many studies have looked at how farmers’ demographic and background characteristics (such as age, gender, education level, work experience, income, and training in pesticide use,) affect their use of protective measures. Of these factors, education level had the greatest influence on farmers’ use of PPE. Many studies have found that farmers’ education level and literacy are key factors in their use of PPE and safe management when handling pesticides [30,62]. Farmers with higher levels of education are more likely to use PPE when handling pesticides [63]. On the other hand, farmers who are illiterate or have low levels of education are at greater risk when working with pesticides and poisons on their farms. This is because they may not be able to read or understand instructions for using pesticides due to low literacy levels, which can lead to increased exposure and poisoning [64].

Also, among the demographic variables, studies showed a positive relationship between age and the use of PPE and PBs. The results of our study were consistent with other study, indicating that there was a positive association between age and the use of PPE [65]. But contrary to our study, other study showed a negative relationship between age and the use of PPE [64], indicating that older farmers use traditional methods for crop cultivation and do not prefer the use of PPE for health.

This research investigated various ecological levels and found that the majority of studies focused on the individual level (such as knowledge and attitudes), the organizational level (such as pesticide training programs), and the interpersonal level (such as influential others). The majority of the training involved working with pesticides and using pest control poisons. There were very few studies conducted at the community level, despite the fact that community-level training is essential for all farmers in developing countries. There were also very few studies conducted at the public policy level, which included government extension services, law enforcement, media coverage, and government attention to providing farmers with access to guidelines, pesticide information resources, and health costs. Policymakers can help farmers by offering appropriate training programs to improve their agricultural skills and knowledge of how to reduce their exposure to pesticides. One way to provide training to farmers is through Farmer Field Schools (FFSs), where farmers can gain access to specialized knowledge [66].

In this systematic review, most studies measured farmers’ knowledge and attitudes towards pesticides and the implementation of personal protective measures. The results showed that farmers’ knowledge and attitudes towards the use of pesticides and the implementation of protective measures were good, and these two constructs were very effective in improving farmers’ protective behaviors. Also, pesticide training programs have been very effective in increasing farmers’ knowledge. Farmers who had more knowledge about pesticides used more PPE and had better PBs. In the study by Yassin et al, farmers reported high levels of knowledge about the impact of pesticides on health and performed most of the necessary protective measures when using pesticides [67].

The results of the study by Öztas showed that knowledge of the safe use of pesticides is very insufficient, and this lack of knowledge negatively affects their quality of life as well as their occupational health and safety. To increase their level of knowledge, appropriate training programs should be arranged [68].

In general, improving knowledge and attitudes is not enough to change the behavior of farmers towards healthy and safe work, as these two are at the individual level. To prevent farmers from experiencing long-term effects when exposed to pesticides, planning should also be done on other levels of the ecological model [69]. It is also necessary in other studies to pay attention to other constructs and factors, as well as to provide training programs as an organizational factor to increase the awareness and attitudes of farmers in developing countries. One notable point in this study was that the experience of working with pesticides and the experience of working on a farm were examined in the information, and in all these studies, farmers who had experience working with pesticides and farming experience had high awareness and attitudes in performing protective measures when working with pesticides [55,70].

Our findings also showed that the majority of studies did not use models and theories to examine behaviors. Only four studies had used a theory or model. Given that changing the behavior of farmers is difficult and many protective recommendations are never adopted by farmers, to improve the behavior of farmers, multiple studies should be conducted with the aim of addressing all levels of the ecological model (individual, interpersonal, organizational, community, and public policy level) and using appropriate models and theories to overcome barriers such as providing necessary financial resources, offering formal training, and access to government extension services.

Most of the studies examined in this systematic review were of fair quality. Similar to our study, another study in this field found that most studies were of low quality [57]. However, in the systematic review by Sapbamrer et al. on the factors affecting the use of PPE and safe pesticide practices, it was shown that most studies were of good quality. This can be explained by the fact that these studies were cross-sectional and received lower scores on some items due to their cross-sectional nature and were classified as low-quality studies [71].

This study had limitations due to the differences in the primary and secondary outcomes measured, as well as the small sample sizes used in the study designs. These factors made it difficult for us to determine an overall effect size or to perform a meta-analysis.

## 5. Conclusion

Research has shown that there are several key factors that influence the use of PPE and safe behaviors when handling pesticides. Among demographic factors, farmers’ education level and age have been found to be important, while among behavioral and psychological-social factors, farmers’ awareness and positive attitudes towards safety measures have been shown to promote the use of protective measures. Environmental factors, such as access to information about pesticides, extension services, formal training programs, and media coverage, can also help minimize exposure to pesticides and should be prioritized in public policy. Extension services provided by the government can play a crucial role in raising awareness about safe pesticide use through training programs that are tailored to farmers’ education levels and regularly updated. Policymakers and governments should prioritize increasing farmers’ knowledge about using fewer toxic pesticides, especially in developing and poor countries. Additionally, the long-term health effects of pesticide exposure and the benefits of protective measures should be taken into account.

## Supporting information

S1 Checklist PRISMA 2009 checklist. (DOC)

S1 Appendix (DOCX)
