# Supplementary material for: Understanding determinants related to farmers’ protective measures towards pesticide exposure: A systematic review
Source: PLoS One. 2024 Feb 15;19(2):e0298450. doi: 10.1371/journal.pone.0298450 (PMC10868758; doi:10.1371/journal.pone.0298450)
Supplement: S1 Checklist — (DOC) [file pone.0298450.s001.doc]

| **Section/topic** | **#** | **Checklist item** | **Reported on page #** |
| --- | --- | --- | --- |
| **TITLE** | | |  |
| Title | 1 | Identify the report as a systematic review, meta-analysis, or both. | Understanding determinants related to farmers' protective measures towards pesticide exposure: a systematic review  (Page 2, title section) |
| **ABSTRACT** | | |  |
| Structured summary | 2 | Provide a structured summary including, as applicable: background; objectives; data sources; study eligibility criteria, participants, and interventions; study appraisal and synthesis methods; results; limitations; conclusions and implications of key findings; systematic review registration number. | Objective: Pesticide poisoning is the main cause of adverse effects and mortality worldwide. Protective measures can reduce the intensity of the effects of pesticides on the health of farmers. Numerous cross-sectional studies have been conducted on the determinants of performing protective measures to reduce exposure to pesticides, but there is no systematic study that comprehensively examines the impact of these factors. Therefore, the aim of this study is to identify existing studies on the determinants of effective protective measures to reduce exposure to pesticides among farmers.  Methods: In this systematic review, studies were obtained from PubMed, Web of Science and Scopus databases using a search strategy that covered articles from the first years of database design to April 20, 2023. The inclusion and exclusion criteria were based on the PICOs criteria. The study included cross-sectional studies that measured the implementation of protective measures using objective or valid subjective tools. The data were extracted and analyzed based on several criteria and ecological levels. The Ecological Model of Health Behavior was used to classify the determinants that affect the performance of protective behaviors. The National Heart, Lung and Blood Institute (NHLBI) has developed a quality assessment tool for studies.  Results**:** A total of 39 studies were ultimately selected for inclusion in this analysis. Many of these studies were conducted in developing countries. The most important factors that have an impact on protective measures include a variety of socio-demographic characteristics (age, gender, level of education, income, farming experience, experience of using pesticides), individual level (knowledge, attitude, risk perception, intention), interpersonal level (subjective norms), organizational level (education), and public policy level (government attention, health costs, governmental extension services). The quality of most studies was fair.  Conclusions: Research indicates that several factors influence the use of personal protective equipment and safe behaviors when handling pesticides. These include farmers’ education level, knowledge, and attitudes towards safety measures. Environmental factors such as access to information, extension services, training programs, and media coverage can also help minimize exposure to pesticides.  (Page 2, The Abstract section)  This project has been approved by the Research and Technology Deputy of Hamadan University of Medical Sciences.  (Page 13, the Acknowledgment section) |
| **INTRODUCTION** | | |  |
| Rationale | 3 | Describe the rationale for the review in the context of what is already known. | Pesticides play a crucial role in controlling harmful or destructive pest species in crops, including insects, weeds, and disease-causing agents. Therefore, the use of pesticides is currently a key strategy in pest management to ensure food supply and distribution worldwide. However, reports indicate that some pesticides pose serious threats to human health and the environment. Several studies have shown that improper use of pesticides can increase the incidence of poisoning, disability, and death associated with pesticide exposure.  Pesticide poisoning is the main cause of adverse health effects and mortality worldwide. It is estimated that the annual incidence of pesticide poisoning among agricultural pesticide users in developing countries is about 18.2 cases per 100,000 people. However, estimating the actual incidence of pesticide poisoning among farmers in developing countries is difficult. Therefore, the use of pesticides has increased health risks for farmers.  The actions such as avoiding the health hazards of pesticides, adopting protective behaviors (PBs), using personal protective equipment (PPE), and appropriate use of pesticides during handling, transportation, mixing, and spraying are recognized as protective measures that can reduce the intensity of the effects of pesticides on the health of farmers. Most farmers do not consider the use of safety measures that can reduce the risk of pesticide poisoning. Therefore, identifying the effective factors on protective measures when using pesticides among farmers is essential.  A review of previous studies in the field of safety shows that various factors can influence protective measures among farmers when faced with pesticide poisoning. Factors such as age, education level, farming experience, perceived risk, awareness, attitudinal and belief variables, perceived barriers, facilitators, health expectations, social norms, emotions, physiological arousal, and intention are among the factors that determine the implementation of protective measures by farmers during pesticide use.  Many farmers who suffer from pesticide poisoning often do not report it due to concerns about losing their job, high costs, and lack of access to health care. Additionally, health care professionals often cannot accurately diagnose pesticide poisoning. Therefore, there is less reporting of pesticide poisoning among farmers. Furthermore, the status of protective measures and their effective determinants in a comprehensive study to reduce poisoning and exposure to pesticides is unclear.  (Page 3, the Introduction section) |
| Objectives | 4 | Provide an explicit statement of questions being addressed with reference to participants, interventions, comparisons, outcomes, and study design (PICOS). | Numerous cross-sectional studies have been conducted on the determinants of performing protective measures to reduce exposure to pesticides, but there is no systematic study that comprehensively examines the impact of these factors. Therefore, the aim of this study is to identify existing studies on the determinants of effective protective measures to reduce exposure to pesticides among farmers.  (Page 3 and 4, last paragraph of the introduction section ) |
| **METHODS** | | |  |
| Protocol and registration | 5 | Indicate if a review protocol exists, if and where it can be accessed (e.g., Web address), and, if available, provide registration information including registration number. | The protocol of this review has been approved by the Research and Technology Deputy of Hamadan University of Medical Sciences (ID number: 140203091801). |
| Eligibility criteria | 6 | Specify study characteristics (e.g., PICOS, length of follow-up) and report characteristics (e.g., years considered, language, publication status) used as criteria for eligibility, giving rationale. | Based on the PRISMA criteria, the inclusion and exclusion criteria for this study were determined.  Inclusion criteria  Study type: Cross-sectional studies were considered for inclusion in this study.  Study topic: Studies that investigated the determinants and factors affecting the use of protective measures (protective behaviors or personal protective equipment) among farmers when dealing with pesticides were examined.  Study population: Farmers who used pesticides in the agricultural sector.  Study outcomes: The outcome of interest was the implementation of protective measures (protective behaviors or use of personal protective equipment), which was measured using objective criteria (observation) or valid subjective tools (self-reporting).  Timeframe: Studies published from the earliest years available in the searched databases were included.  Language: Studies published in all languages were considered.  Location: Studies conducted in all countries and on both large and small farms were reviewed.  Exclusion criteria  The absence of appropriate format in articles, inaccurate mention of sample size and characteristics, study design, special studies (such as letters to the editor, comments), review articles, structured reviews, meta-analyses, conference presentations, qualitative studies, various intervention studies, cohort and case-control studies, as well as agricultural and floricultural workers, greenhouse workers, women, and children in rural areas, were considered as exclusion criteria.  (Pages 4 and 5, the Methods section) |
| Information sources | 7 | Describe all information sources (e.g., databases with dates of coverage, contact with study authors to identify additional studies) in the search and date last searched. | The articles were obtained from the PubMed, Web of Science, and Scopus databases using a search strategy developed by one of the researchers. In this search strategy, the keywords present in Appendix A were used. The search strategy was for articles searched from the first years of database design to April 20, 2023. Modifications were made to this search strategy according to the differences in search capabilities of each database.  (Page 4, the Methods section) |
| Search | 8 | Present full electronic search strategy for at least one database, including any limits used, such that it could be repeated. | Search strategy for PubMed  Agricultur* OR agriculture OR farm OR farms OR farmer* OR farming OR "farm worker" OR "farm workers" OR "farmworker" OR "farmworkers" OR greenhous* OR orchard* OR "crop production" OR harvesting OR horticultur* OR horticulture OR agronom* OR mix* OR work OR occupation OR "crops, agricultural" OR "agricultural workers' diseases" OR gardener OR "agricultural worker" OR "fruit grower" OR orchardist OR grower OR cultivator OR planter  AND  Pesticid* OR pesticides OR fungicid* OR herbicid* OR organophosphate OR biocides OR herbicide OR insecticid* OR carbamat* OR carbamates OR pyrethrins OR molluscacid* OR rodenticid* OR poison* OR "pesticide exposure" OR fungicide OR fumigant  AND  Determinant* OR Determinants OR anticipation* OR factors* OR factor OR predictive* OR forecasting OR “risk factors” OR forecast* OR prediction*  AND  “Cross–Sectional” OR “descriptive-analytical” OR "Cross Sectional Studies" OR “Cross Sectional” OR "Cross-Sectional Study" OR "Cross Sectional Study" OR "Cross-Sectional Studies" OR "Cross Sectional Analysis" OR "Cross-Sectional Analysis" OR "Cross Sectional Analyses" OR "Cross-Sectional Analyses" OR "Cross-Sectional Survey" OR "Cross Sectional Survey" OR "Prevalence Studies" OR "Prevalence Study"  (Appendix A) |
| Study selection | 9 | State the process for selecting studies (i.e., screening, eligibility, included in systematic review, and, if applicable, included in the meta-analysis). | All retrieved articles from the selected databases were organized and screened using EndNote software. The search for articles from the databases was conducted by one of the researchers to minimize errors.  After removing any duplicates, the titles and abstracts of the articles are assessed to determine their eligibility. The full text of eligible articles and relevant data are then extracted and recorded in a data extraction table. This process is carried out independently by two researchers. In case of disagreement on the remaining articles, a third researcher will review and provide their opinion to resolve the conflict. Additionally, the list of references used in the remaining and relevant articles is also reviewed to ensure completeness.  (Page 5, the methods section) |
| Data collection process | 10 | Describe method of data extraction from reports (e.g., piloted forms, independently, in duplicate) and any processes for obtaining and confirming data from investigators. | The data were extracted based on the following criteria: first author's name, year of study, location of study, study objective, journal name, sampling technique, sample size, determinants examined in the studies, model or theory used in the studies, and study results. Additionally, the determinants were evaluated as independent variables at five ecological levels, and their impact on the dependent variable, which includes performing protective behaviors or using personal protective equipment, was examined in the study. Demographic and background characteristics were also examined in the data extraction process.  (Page 5, the Methods section) |
| Data items | 11 | List and define all variables for which data were sought (e.g., PICOS, funding sources) and any assumptions and simplifications made. | -- |
| Risk of bias in individual studies | 12 | Describe methods used for assessing risk of bias of individual studies (including specification of whether this was done at the study or outcome level), and how this information is to be used in any data synthesis. | The quality assessment tool for cross-sectional studies available from National Heart, Lung and Blood Institute (NHLBI), USA was used for quality assessment. This tool consists of 14 criteria that cover various aspects of the study design, methods, and analysis, such as the research question, the study population, the exposure and outcome measures, the confounding variables, and the statistical methods. For each criterion, researchers can answer yes, no, or other (CD, NR, NA), depending on whether the study met the criterion, did not meet the criterion, or they cannot determine, not reported, or not applicable. Researchers can also provide a quality rating (good, fair, or poor) for each study based on their overall assessment of the criteria. Quality is rated on a scale of 0 to 2, where 0 represents poor quality, 1 represents fair quality, and 2 represents good quality. The rating is determined based on the number of questions answered correctly out of a total of 14 questions. A score of 0-4 out of 14 questions corresponds to a rating of 0 (poor), a score of 5-10 out of 14 questions corresponds to a rating of 1 (fair), and a score of 11-14 out of 14 questions corresponds to a rating of 2 (good). NA stands for “not applicable” and NR stands for “not reported”.  (Page 6, the Methods section) |
| Summary measures | 13 | State the principal summary measures (e.g., risk ratio, difference in means). | Details of the included studies are reported in Tables 1 and Fig 1 to 3. Due to the differences in interventions and target outcomes, no meta-analysis was attempted on the included studies.  (Page 5, the Methods section) |
| Synthesis of results | 14 | Describe the methods of handling data and combining results of studies, if done, including measures of consistency (e.g., I^2^) for each meta-analysis. | In this study, the Ecological Model of Health Behavior (EMHB) was used to classify determinants that affect the performance of protective behaviors. The first level of the model is the individual level, which is related to the knowledge, attitudes, and skills that are directly related to the individual. The second level is the interpersonal level, which includes the exchanges and interactions within an individual's network. This includes primary relationships, such as family and close friends, as well as secondary groups that are larger and more extensive. The third level is the organizational level focused on social institutions that act as official authorities and provide public and accepted goals. The fourth level is the community level, which includes relationships that organizations create with each other. These relationships are often found in coalitions. Finally, the fifth level is the policy and public policy level that is adopted by local and national governments. Ideally, when integrating and using this model, all five levels are taken into account.  (Pages 5 and 6, the Methods section) |

Page 1 of 2

| **Section/topic** | **#** | **Checklist item** | **Reported on page #** |
| --- | --- | --- | --- |
| Risk of bias across studies | 15 | Specify any assessment of risk of bias that may affect the cumulative evidence (e.g., publication bias, selective reporting within studies). | -- |
| Additional analyses | 16 | Describe methods of additional analyses (e.g., sensitivity or subgroup analyses, meta-regression), if done, indicating which were pre-specified. | -- |
| **RESULTS** | | |  |
| Study selection | 17 | Give numbers of studies screened, assessed for eligibility, and included in the review, with reasons for exclusions at each stage, ideally with a flow diagram. | Through electronic search engines and strategies, a total of 895 studies were identified. During the initial evaluation of duplicate and title, 817 of these studies were excluded as they did not meet the inclusion criteria. Twenty nine articles were excluded during the evaluation of abstracts, resulting in a total of 49 studies for full-text assessment. After further assessment, a final selection of 39 studies was made for inclusion in the analysis.  (Page 6, the Results section) |
| Study characteristics | 18 | For each study, present characteristics for which data were extracted (e.g., study size, PICOS, follow-up period) and provide the citations. | Studies have been conducted on farmers from 2008 until 2023.  The articles included in the analysis were based on studies conducted in the Australia (n = 1), Iran (n= 4), Canada (n= 1), China (n= 1), Uganda (n= 3), Thailand (n= 3), Egypt (n= 1), Brazil (n= 2), Nepal (n= 3), Pakistan (n= 3), Italy (n= 1), Turkey (n= 1), India (n= 1), Malaysia (n= 1), Ghana (n= 1), Morocco (n= 2), Nigeria (n= 2), Vietnam (n= 1), Philippine (n= 1), Costa Rica and Uganda (n= 1), and Ethiopia (n= 5). Among the 39 articles obtained in this study, sampling techniques included simple random sampling (n=12), quota sampling (n=3), systematic random sampling (n=2), stratified random sampling (n=1), convenience sampling (n=3), purposive sampling (n=1), snowball sampling (n=4), not clear (n=1), and some were multi stage sampling (n=12). The total number of samples was low. Less than 350 samples were used in twenty three studies.  *Theoretical framework usage*  A theoretical framework was applied in only four articles (10.2%) that were included. The integrated agent centered (IAC) framework, precede-proceed framework, trans-theoretical model (TTM), and theory of planned behavior (TPB) were the theories that were used. *Categorization of determinants* 1) Demographic examined in studies  **All studies had taken into account demographic characteristics more or less.** Except for two studies, other studies had taken age as one of the demographic characteristics into account. Twenty five articles had considered gender. The level of education had been examined for the participants in Twenty nine articles. Also, the marital status of the participants had been investigated in seventeen studies. The income status of the farmers had also been asked in eleven articles. The years of farming of the farmers was another characteristic that was examined in eight studies. The duration of exposure with pesticides and the years of spraying crops were other items that had been asked from the farmers in twelve studies. The ownership of the orchard or agricultural land was another item that had been investigated in four studies. Also, race/ ethnicity had been reported in three studies and religion in two articles. The size of the farm and family size were also another factor that had been asked from the farmers. The consumption of smoking and alcohol by the farmers had also been reported in six studies. The history of having a child and a pregnant woman had also been examined in two studies. The type of crops in two studies and the medical history of the farmers in one study had been considered.  2) Ecological Model of Health Behavior examined in studies  Individual level: Almost all studies except for three studies had considered knowledge as a determinant of protective measures. Also, most studies had examined attitude as a determinant of performing protective measures. Risk perception/ perceived risk was the next determinant that had been investigated in five studies out of 39 studies. The intention in two studies was considered to see what relationship it had with protective actions. The locus of control, confidence, eagerness to learn, levels of readiness to change, health effects, perceived importance, decision making, and health consequences each were investigated in one study. The perceived behavioral control, perceived susceptibility, and perceived severity were also only investigated in one study. The determinants of perceived barriers, facilitators, social outcome expectations, health outcome expectations, expected value of consequences, affect, physiological arousal, and habit, in a study were investigated.  Interpersonal level: Reinforcing factors in one study were considered as a determinant of protective measures. Also, subjective norms were considered in two studies. Social norm was also investigated in another study.  Organizational level: In eight studies, education to farmers about pesticides and exposure to them through governmental and private organizations were considered as an organizational factor. Enabling factors and access to materials were also each investigated in one study.  Community level: At this level, only studies had examined two social and environmental factors that could affect protective measures.  Public policy level: The determinants of government attention, media coverage, enforcement of laws, and guideline access, in a study were investigated. In another study, governmental extensions services were considered for the impact on protective measures in exposure to pesticides. Also, in one study, health costs and in another study, the source of information dissemination were examined.  (Pages 6 to 8, the Results section) |
| Risk of bias within studies | 19 | Present data on risk of bias of each study and, if available, any outcome level assessment (see item 12). | Please see Table 1  The quality of most studies was fair, and only five studies had good quality in the assessment by researchers.  (Page 10, the Results section) |
| Results of individual studies | 20 | For all outcomes considered (benefits or harms), present, for each study: (a) simple summary data for each intervention group (b) effect estimates and confidence intervals, ideally with a forest plot. | Please see Table 1 and Fig 2 and 3 |
| Synthesis of results and Risk of bias across studies | 21 | Present results of each meta-analysis done, including confidence intervals and measures of consistency.  Present results of any assessment of risk of bias across studies (see Item 15). | In the following, the variables that had a positive effect on protective measures and had increased the performance of these actions are mentioned and the determinants that had no relationship are not explained.  1) Demographic examined in studies  The age of farmers had a statistically significant association with protective behavior to reduce exposure to pesticides. In three studies, gender had a statistically significant association with protective measures. Also, in thirteen studies, the results showed that the level of education of farmers had a statistically significant association with protective measures. The income of farmers also showed a statistically significant association with protective measures in two studies. In three studies, farming experience and in three studies, experience of using pesticides showed a statistically significant association with protective measures. In one study, ethnicity and in another study, alcohol consumption had a significant statistical relationship with protective measures.  2) Ecological Model of Health Behavior examined in studies  Individual level: Out of 36 studies that examined knowledge, it was found that 35 studies had a statistically significant association with protective measures. Out of 25 studies that worked on attitude, it was found that in three studies, no statistically significant association with protective measures was reported. All five studies that used risk perception/ perceived risk reported that this construct could have a positive effect on protective measures. Both studies that examined the effect of intention on protective measures reported a statistically significant association between the two. The constructs of locus of control, confidence, eagerness to learn, levels of readiness to change, health effects, decision making, perceived importance, perceived behavioral control, perceived susceptibility, perceived severity, physiological arousal, habit perceived barriers, facilitators, and health consequences had a statistically significant association with protective measures.  Interpersonal level: The constructs of reinforcing factors had a statistically significant association with protective measures. Also, both studies that examined the effect of subjective norms on protective measures reported a statistically significant association between the two.  Organizational level: Out of eight studies that farmers had considered the factor of farmer education by organizations, seven studies confirmed the association between acquiring these educations and protective measures. In addition, the access to materials had a statistically significant association with protective measures.  Public policy level: The determinants of government attention, media coverage, enforcement of laws, and guideline access, in a study were investigated, governmental extensions services, health costs, and source of information dissemination had a statistically significant association on protective measures in exposure to pesticides.  (Pages 9 to 10, the Results section) |
| Additional analysis | 23 | Give results of additional analyses, if done (e.g., sensitivity or subgroup analyses, meta-regression [see Item 16]). | -- |
| **DISCUSSION** | | |  |
| Summary of evidence | 24 | Summarize the main findings including the strength of evidence for each main outcome; consider their relevance to key groups (e.g., healthcare providers, users, and policy makers). | In this study, after careful examination of articles by researchers, 39 studies were identified in the field of determining the effective determinants of protective measures to reduce exposure to pesticides. In this systematic review, most studies were conducted in developing countries. In developing countries, farmers are at high risk of exposure to pesticides due to having small-scale agricultural land and gardens, as well as a lack of protective equipment, climatic conditions, and excessive use of pesticides. Contrary to our study, the systematic study by Afshari and et al (2021), which was conducted on interventional studies in the field of reducing exposure to pesticides in farmers, showed that most studies were conducted in developed countries, including the United States. Wiedemann and colleagues in a systematic study showed that in developing countries, extension services cannot reach the entire agricultural community due to insufficient budget. In wealthy and developed countries, strict regulations have been imposed for the use of pesticides, and exposure to toxic chemicals is prohibited or limited for farmers. While in developing countries, regulations and supervision of the use of pesticides are challenging and not implemented, and farmers are exposed to a large extent to toxic and vulnerable substances. So that, 1% increase in crop production per hectare is associated with a 1.8% increase in pesticide use per hectare. However, as countries reach higher levels of economic development, the growth in pesticide use intensity decreases.  The current study found that many studies have looked at how farmers’ demographic and background characteristics (such as age, gender, education level, work experience, income, and training in pesticide use,) affect their use of protective measures. Of these factors, education level had the greatest influence on farmers’ use of personal protective equipment. Many studies have found that farmers’ education level and literacy are key factors in their use of PPE and safe management when handling pesticides. Farmers with higher levels of education are more likely to use personal protective equipment when handling pesticides. On the other hand, farmers who are illiterate or have low levels of education are at greater risk when working with pesticides and poisons on their farms. This is because they may not be able to read or understand instructions for using pesticides due to low literacy levels, which can lead to increased exposure and poisoning.  Also, among the demographic variables, studies showed a positive relationship between age and the use of personal protective equipment and protective behaviors. The results of our study were consistent with other study, indicating that there was a positive association between age and the use of personal protective equipment. But contrary to our study, other study showed a negative relationship between age and the use of personal protective equipment, indicating that older farmers use traditional methods for crop cultivation and do not prefer the use of personal protective equipment for health.  This research investigated various ecological levels and found that the majority of studies focused on the individual level (such as knowledge and attitudes), the organizational level (such as pesticide training programs), and the interpersonal level (such as influential others). The majority of the training involved working with pesticides and using pest control poisons. There were very few studies conducted at the community level, despite the fact that community-level training is essential for all farmers in developing countries. There were also very few studies conducted at the public policy level, which included government extension services, law enforcement, media coverage, and government attention to providing farmers with access to guidelines, pesticide information resources, and health costs. Policymakers can help farmers by offering appropriate training programs to improve their agricultural skills and knowledge of how to reduce their exposure to pesticides. One way to provide training to farmers is through Farmer Field Schools (FFSs), where farmers can gain access to specialized knowledge.  In this systematic review, most studies measured farmers’ knowledge and attitudes towards pesticides and the implementation of personal protective measures. The results showed that farmers’ knowledge and attitudes towards the use of pesticides and the implementation of protective measures were good, and these two constructs were very effective in improving farmers’ protective behaviors. Also, pesticide training programs have been very effective in increasing farmers’ knowledge. Farmers who had more knowledge about pesticides used more personal protective equipment and had better protective behaviors. In the study by Yassin et al, farmers reported high levels of knowledge about the impact of pesticides on health and performed most of the necessary protective measures when using pesticides.  The results of the study by Öztas showed that knowledge of the safe use of pesticides is very insufficient, and this lack of knowledge negatively affects their quality of life as well as their occupational health and safety. To increase their level of knowledge, appropriate training programs should be arranged.  In general, improving knowledge and attitudes is not enough to change the behavior of farmers towards healthy and safe work, as these two are at the individual level. To prevent farmers from experiencing long-term effects when exposed to pesticides, planning should also be done on other levels of the ecological model. It is also necessary in other studies to pay attention to other constructs and factors, as well as to provide training programs as an organizational factor to increase the awareness and attitudes of farmers in developing countries. One notable point in this study was that the experience of working with pesticides and the experience of working on a farm were examined in the information, and in all these studies, farmers who had experience working with pesticides and farming experience had high awareness and attitudes in performing protective measures when working with pesticides.  Our findings also showed that the majority of studies did not use models and theories to examine behaviors. Only four studies had used a theory or model. Given that changing the behavior of farmers is difficult and many protective recommendations are never adopted by farmers, to improve the behavior of farmers, multiple studies should be conducted with the aim of addressing all levels of the ecological model (individual, interpersonal, organizational, community, and public policy level) and using appropriate models and theories to overcome barriers such as providing necessary financial resources, offering formal training, and access to government extension services.  Most of the studies examined in this systematic review were of fair quality. Similar to our study, another study in this field found that most studies were of low quality. However, in the systematic review by Sapbamrer et al. on the factors affecting the use of personal protective equipment and safe pesticide practices, it was shown that most studies were of good quality. This can be explained by the fact that these studies were cross-sectional and received lower scores on some items due to their cross-sectional nature and were classified as low-quality studies  (Pages 10 to 12, the Discussion section) |
| Limitations | 25 | Discuss limitations at study and outcome level (e.g., risk of bias), and at review-level (e.g., incomplete retrieval of identified research, reporting bias). | This study had limitations due to the differences in the primary and secondary outcomes measured, as well as the small sample sizes used in the study designs. These factors made it difficult for us to determine an overall effect size or to perform a meta-analysis.  (Page 13, the Discussion section) |
| Conclusions | 26 | Provide a general interpretation of the results in the context of other evidence, and implications for future research. | Research has shown that there are several key factors that influence the use of personal protective equipment and safe behaviors when handling pesticides. Among demographic factors, farmers’ education level and age have been found to be important, while among behavioral and psychological-social factors, farmers’ awareness and positive attitudes towards safety measures have been shown to promote the use of protective measures. Environmental factors, such as access to information about pesticides, extension services, formal training programs, and media coverage, can also help minimize exposure to pesticides and should be prioritized in public policy. Extension services provided by the government can play a crucial role in raising awareness about safe pesticide use through training programs that are tailored to farmers’ education levels and regularly updated. Policymakers and governments should prioritize increasing farmers’ knowledge about using fewer toxic pesticides, especially in developing and poor countries. Additionally, the long-term health effects of pesticide exposure and the benefits of protective measures should be taken into account.  (Pages 16, the Conclusion section) |
| **FUNDING** | | |  |
| Funding | 27 | Describe sources of funding for the systematic review and other support (e.g., supply of data); role of funders for the systematic review. | This work was supported by Hamadan University of Medical Sciences [reference number: 140203091801]. |

*From:*  Moher D, Liberati A, Tetzlaff J, Altman DG, The PRISMA Group (2009). Preferred Reporting Items for Systematic Reviews and Meta-Analyses: The PRISMA Statement. PLoS Med 6(7): e1000097. doi:10.1371/journal.pmed1000097

For more information, visit: **www.prisma-statement.org**.
