# Supplementary material for: Understanding determinants related to farmers’ protective measures towards pesticide exposure: A systematic review
Source: PLoS One. 2024 Feb 15;19(2):e0298450. doi: 10.1371/journal.pone.0298450 (PMC10868758; doi:10.1371/journal.pone.0298450)
Supplement: S1 Appendix — (DOCX) [file pone.0298450.s002.docx]

**Appendix A**

Agricultur* OR agriculture OR farm OR farms OR farmer* OR farming OR "farm worker" OR "farm workers" OR "farmworker" OR "farmworkers" OR greenhous* OR orchard* OR "crop production" OR harvesting OR horticultur* OR horticulture OR agronom* OR mix* OR work OR occupation OR "crops, agricultural" OR "agricultural workers' diseases" OR gardener OR "agricultural worker" OR "fruit grower" OR orchardist OR grower OR cultivator OR planter

AND

Pesticid* OR pesticides OR fungicid* OR herbicid* OR organophosphate OR biocides OR herbicide OR insecticid* OR carbamat* OR carbamates OR pyrethrins OR molluscacid* OR rodenticid* OR poison* OR "pesticide exposure" OR fungicide OR fumigant

AND

Determinant* OR Determinants OR anticipation* OR factors* OR factor OR predictive* OR forecasting OR “risk factors” OR forecast* OR prediction*

AND

“Cross–Sectional” OR “descriptive-analytical” OR "Cross Sectional Studies" OR “Cross Sectional” OR "Cross-Sectional Study" OR "Cross Sectional Study" OR "Cross-Sectional Studies" OR "Cross Sectional Analysis" OR "Cross-Sectional Analysis" OR "Cross Sectional Analyses" OR "Cross-Sectional Analyses" OR "Cross-Sectional Survey" OR "Cross Sectional Survey" OR "Prevalence Studies" OR "Prevalence Study"
